# Aberrant Expression of Clock Gene Period1 and Its Correlations with the Growth, Proliferation and Metastasis of Buccal Squamous Cell Carcinoma

**DOI:** 10.1371/journal.pone.0055894

**Published:** 2013-02-06

**Authors:** Ningbo Zhao, Kai Yang, Genling Yang, Dan Chen, Hong Tang, Dan Zhao, Chunrong Zhao

**Affiliations:** 1 Department of Oral and Maxillofacial Surgery, The First Affiliated Hospital of Chongqing Medical University, Chongqing, China; 2 Chongqing Medical University Laboratory Animal Center, Chongqing, China; University of Saarland Medical School, Germany

## Abstract

Period1 (PER1) is an important core clock gene, which regulates normal cell proliferations and physiological rhythms of human beings. Recent studies have showed aberrant expressions and altered rhythms of PER1 were highly correlated to the carcinogenesis and development of malignant tumors. However, there is no study on the correlation of aberrant expressions and altered rhythms of PER1 with the growth, proliferation and metastasis of buccal squamous cell carcinoma (BSCC). In this study, PER1 and MMP-2 expression in the cancerous and adjacent noncancerous tissues of 38 patients with BSCC and its correlations with patients' clinical pathologic characteristics were investigated. A mouse model of BSCC was also established and mice were sacrificed at 4 different time points in a period of 24 hours. Xenografted tumor weight, proliferation index (PI), and mitotic index (MI) of tumors in the 4 time groups were detected. Results showed that PER1 expression was significantly down-regulated in cancerous tissues of patients with BSCC (*P*<0.05). PER1 expression was significantly down-regulated in patients of T3∼T4 staging and those with lymph node metastasis compared to that of T1∼T2 staging and those without lymph node metastasis (*P*<0.05), respectively. PER1 mRNA expression, MI and tumor weight had significant differences among the 4 time groups, which PI all confirmed to circadian rhythms. MI, PI, MMP-2 mRNA and tumor weight had negative correlation with PER1 mRNA expression. Peak value of PER1 mRNA expression and trough values of MI, PI and tumor weight all appeared in middle activity phase, whereas trough value of PER1 mRNA expression and peak values of MI, PI and tumor weight all occurred in middle rest phase. Our study suggested that aberrant expression of PER1 had significant correlation with the growth, proliferation and metastasis of BSCC and it might act as an anti-oncogene.

## Introduction

In humans, various physiological processes, such as body temperature, heart rate, blood pressure, hormone secretion and cell metabolism, show circadian rhythm [1], [2]. Circadian rhythm, one of the basic characteristics of vital movements of living subjects, is controlled by a special circadian system in vivo [3]. This special circadian system is composed of a series of clock genes. To date, at least nine known core clock genes are found [4], including period1 (PER1), period2 (PER2), period3 (PER3), CLOCK, cryptochrome1 (CRY1), cryptochrome2 (CRY2), BMAL1, casein kinase1 epsilon (CSNK1E) and timeless(TIM). These clock genes regulate proliferation, secretion, metabolism of normal cells and circadian rhythm of living subjects, through changing their own expression levels and rhythmic patterns [5]–[9]. The basic characteristic of malignant tumor is its uncontrolled and disordered cell proliferation [10]. Therefore, aberrant expressions of these clock genes can change proliferation and rhythm of normal cells, which can easily lead to carcinogenesis. Studies in recent years have validated that aberrant expressions and altered rhythms of clock genes were highly related to the carcinogenesis, development, therapeutic effects and prognosis of many cancers [11]–[17].

PER1, an important core clock gene, involves in the regulation of normal cell proliferations and physiological rhythms of human beings. Its function is to regulate physiological rhythms, cell cycle and DNA damage response of normal living subjects [1], [2], [5], [11]. Previous studies have showed that aberrant expressions and altered rhythms of PER1 were highly linked to the carcinogenesis and development of malignant tumors, such as prostate cancer, colon cancer, leukocythemia and breast cancer et al [12]–[17]. However, the expression level, function and circadian rhythm of PER1 altered greatly in different tumor types [5], [12], [13], [15], [17]–[20]. Gery et al [5] showed that PER1 was down-regulated in prostate and colon cancers and played a pro-apoptotic and cancer-suppressive role, while Sato et al [20] demonstrated that PER1 was up-regulated in pancreatic and liver cancers and exerted an anti-apoptotic and cancer-promotive effect. Until now, we have not found related reports about variations of PER1 expression in the tumorigenesis and development of BSCC.

Oral cancer, with the incidence about 0.47∼3.22 per million, accounts for about 2% of the systemic malignancies. And more than ninety percent of the oral cancers correspond to squamous cell carcinomas [21], [22]. Although great progress has been achieved in various therapeutic methods in recent decades, the 5-year overall survival rate for post-treatmented patients with oral squamous cell carcinoma (OSCC) is only 55%∼60% [22]. Therefore, it is significant to explore new methods for the treatment of OSCC. BSCC is the most common oral cancer. In this study, we have investigated PER1 and MMP-2 protein expression in the cancerous tissues of 38 patients with BSCC and their correlations with patients' clinical pathologic characteristics. And we have established a mouse model of BSCC to detect the circadian expressions of PER1 and MMP-2 in tumors. We also analyzed the correlations of PER1 expression and tumor growth, proliferation and tumor invasion and metastasis, trying to, from the perspective of the expression and altered rhythm of PER1, provide new ideas and methods for further investigation of the carcinogenesis, development, and individualized treatment of OSCC.

## Results

### PER1 and MMP-2 protein expressions in cancerous and adjacent noncancerous tissues of patients with BSCC and their correlations with patients' clinical pathologic characteristics

**Figure 1:**
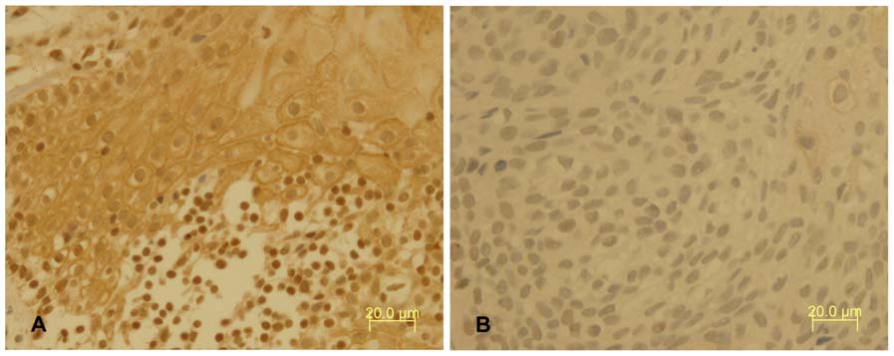
Immunohistochemical staining of PER1 protein expression in tissues of patients with BSCC. A: PER1 was strongly positive in the adjacent noncancerous tissues. B: PER1 was positive or weakly positive in cancerous tissues (magnification, ×400).

**Figure 2:**
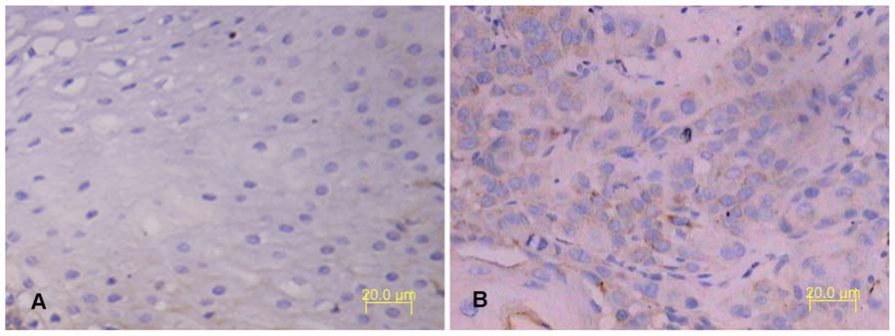
Immunohistochemical staining of MMP-2 protein expression in tissues of patients with BSCC. A: MMP-2 was negative in the adjacent noncancerous tissues. B: MMP-2 was positive in cancerous tissues (magnification, ×200).

**Figure 3:**
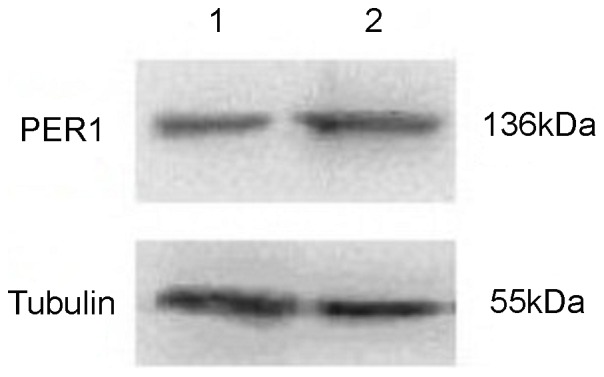
Western blot analysis of PER1 protein expression in tissues of patients with BSCC. The level of Tubulin in each sample is detected as an internal control. One representative of three independent experiments with similar results is shown. Lane 1: PER1 protein expression in cancerous tissues. Lane 2: PER1 protein expression in adjacent noncancerous tissues.

IHC results showed PER1 protein expressions in cancerous and adjacent noncancerous tissues of patients with BSCC were 84.2% (32/38) and 100% (38/38), respectively. The difference had statistical significance (*P*<0.05). In adjacent noncancerous tissues PER1 protein expression was strongly positive, which mainly stained in nucleus and secondly stained in cytoplasm, while in cancerous tissues it was positive or weakly positive, which stained both in nucleus and cytoplasm (see Fig. 1). MMP-2 protein expressions in cancerous and adjacent noncancerous tissues of patients with BSCC were 76.3%(29/38) and 7.9%(3/38), respectively. The difference had statistical significance (*P*<0.05). MMP-2 protein stained mostly in cytoplasm and slightly in intercellular substance (see Fig. 2). Western Blot analysis of PER1 protein expressions in cancerous and adjacent noncancerous tissues of patients with BSCC were showed in Figure 3. PER1 protein expression was down-regulated in cancerous tissues compared to that in adjacent noncancerous tissues.

PER1 and MMP-2 protein expression was significantly correlated with clinical stage. Further analysis showed PER1 protein expression was up-regulated in I∼II staging compared to that in III∼IV staging. The difference had statistical significance (*P*<0.05); PER1 protein expression was significantly up-regulated in patients of T1∼T2 staging and those without lymph node metastasis compared to that of T3∼T4 staging and those with lymph node metastasis(P<0.05), respectively (see Table 1). However, MMP-2 protein expression and its correlations with patients' clinical pathologic characteristics was opposite to that of PER1 (see Table 1). PER1 and MMP-2 protein expression in cancerous tissues had no significant correlation with patients' age, sex, smoking status, alcohol use status, areca nut chewing and cancer differentiation (*P*>0.05, see Table 1). These results suggested that down-regulated PER1 expression and up-regulated MMP-2 expression were correlated with more advanced cancer stages in BSCC.

**Table 1:** PER1 and MMP-2 expressions in cancerous tissues of patients with BSCC and their correlations with patients' clinical pathologic characteristics.

		Protein expression				
		Per1		MMP-2		
Parameters	Cases	Negative	Positive	Negative	Positive	P value
**Tissue type**						
**Cancerous tissue**	38	6	32	9	29	0.025^a^
**Adjacent noncancerous tissues**	38	0	38	35	3	0.000^b^
**Age**						
**≥60**	9	1	8	2	7	0.869^a^ *
**≤45∼<60**	19	3	16	4	15	0.859^b^ *
**<45**	10	2	8	3	7	
**Sex**						
**Female**	16	3	13	4	12	0.682^a^
**Male**	22	3	19	5	17	1.000^b^
**Smoking status**						
**Never smoker**	10	3	17	4	16	0.480^a^ *
**Ex-smoker**	5	0	5	2	3	0.641^b^ *
**Current smoker**	13	3	10	3	10	
**Alcohol use status**						
**Never drinker**	18	3	16	5	13	0.966^a^ *
**Ex-drinker**	8	1	7	1	7	0.693^b^ *
**Current drinker**	12	2	10	3	9	
**Areca nut chewing**						
**Never chewing**	30	5	25	4	26	0.724^a^ *
**Ex- chewing**	3	0	3	1	2	0.907^b^ *
**Current chewing**	5	1	4	4	1	
**Differentiation**						
**Well**	10	2	8	3	7	0.901^a^ *
**Moderately**	20	3	17	4	16	0.828^b^ *
**Poorly**	8	1	7	2	6	
**T staging**						
**T1+T2**	16	0	16	7	9	0.030^a^
**T3+T4**	22	6	16	2	20	0.021^b^
**Lymph node metastasis**						
**No**	26	1	25	9	17	0.008^a^
**Yes**	12	5	7	0	12	0.036^b^
**Clinical stage**						
**I+II**	20	0	20	9	11	0.007^a^
**II+IV**	18	6	12	0	18	0.001^b^

a, b P value of PER1 and MMP-2 protein expressions, respectively.

* Statistical analyses were performed by the Pearson χ^2^ test.

### The expression and circadian variation of PER1 and MMP-2 in tumors of tumor bearing mice

**Figure 4:**
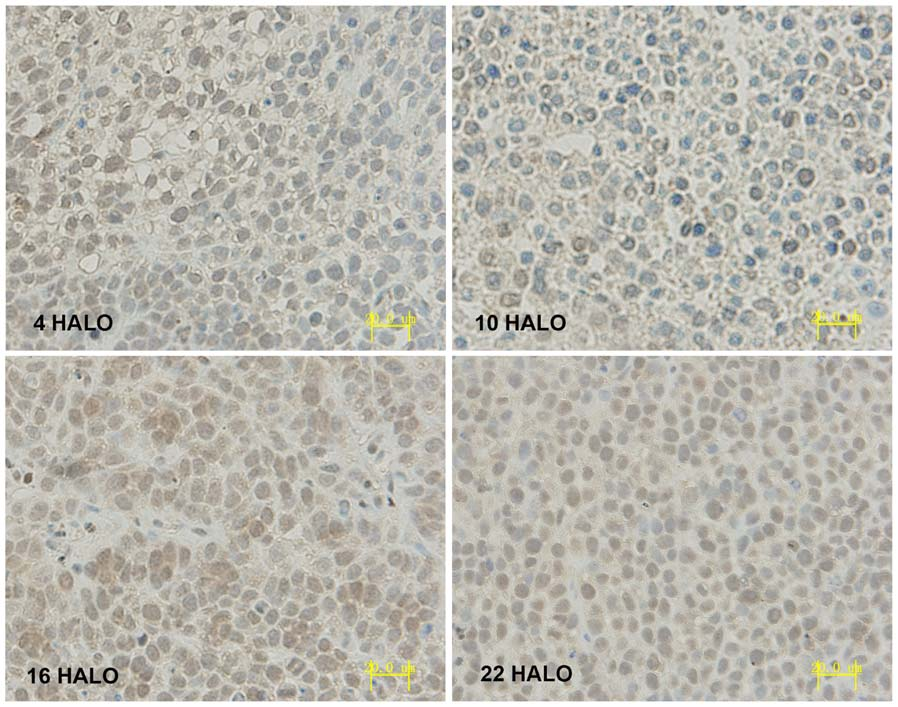
Immunohistochemical staining of PER1 protein expression in tumors of tumor bearing mice. Highest PER1 staining was seen in tumors resected at 16 HALO, while tumors resected at 10 HALO showed the minimum PER1 staining (magnification, ×400).

**Figure 5:**
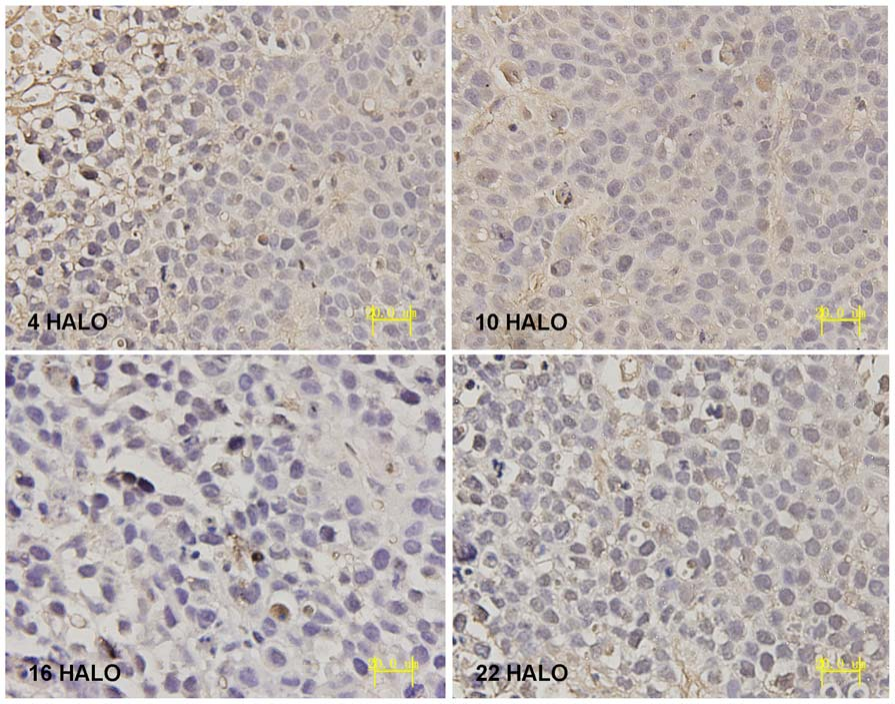
Immunohistochemical staining of MMP-2 protein expression in tumors of tumor bearing mice. Minimum MMP-2 staining was seen in tumors resected at 16 HALO, while tumors resected at 10 HALO showed the highest MMP-2 staining (magnification, ×400).

**Figure 6:**
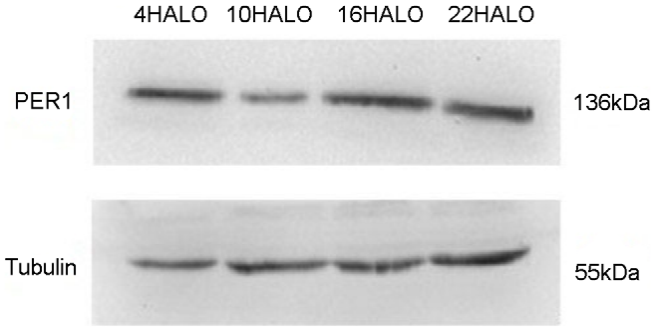
Western blot analyses of PER1 protein expressions of 4 time groups of tumor bearing mice. The level of Tubulin in each sample is detected as an internal control. One representative of three independent experiments with similar results is shown.

IHC results displayed that PER1 and MMP-2 protein was intensely stained in tumors had harvested at 4 HALO, 10 HALO, 16 HALO and 22 HALO. PER1 protein located both in nucleus and cytoplasm (see Fig. 4), MMP-2 protein located in cytoplasm(see Fig. 5). Highest PER1 staining and minimum MMP-2 staining was seen in tumors resected at 16 HALO, whereas tumors resected at 10 HALO show the minimum PER1 staining and highest MMP-2 staining. Western blot analysis showed PER1 protein expressions in tumors among 4 time groups. (see Fig. 6), among which maximum PER1 protein expression was seen in tumors resected at 16 HALO, while tumors resected at 10 HALO showed the minimum PER1 protein expression.

**Figure 7:**
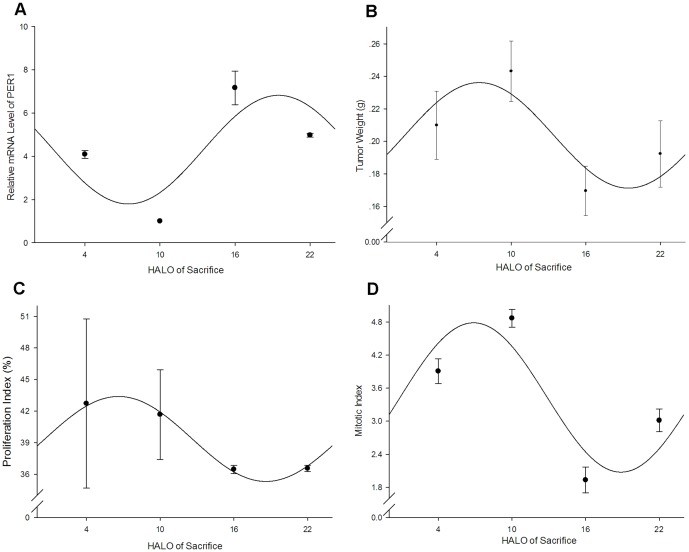
Cosine fitted curves of the circadian variations of PER1 mRNA expression, tumor weight, PI, MI of tumor bearing mice. A: relative PER1 mRNA expression, the average of the lowest relative PER1 mRNA amount was set as 1. β-actin is detected as an internal control; B: tumor weight; C: PI of tumor cells; D: MI of tumor cells. Points, mean with 24 h cosine fitted curves; bars, SD. Note that there is a break in the vertical axis.

The result of real-time RT-PCR indicated that the relative PER1 mRNA expressions of 4 time groups had significant difference (F = 121.24, P<0.01, listed in Table 2). The single cosine test confirmed that PER1 mRNA expressions obeyed a circadian rhythm (F = 7.67, P<0.05). Cosine fitted curve of relative PER1 mRNA expressions was shown in Figure 7A, the peak value of which occurred at 19.48 HALO (the corresponding relative expression was 6.82), while the trough value occurred at 7.64 HALO (the corresponding relative expression was 1.79). The relative MMP-2 mRNA expressions of 4 time groups had significant difference (F = 1723.65, P<0.01, see Table 2). But the single cosine test confirmed that MMP-2 mRNA expressions did not obey a circadian rhythm (F = 2.40, P = 0.25).

**Table 2:** Circadian variations of relative PER1 and MMP-2 mRNA expression, tumor growth and proliferation of tumor bearing mice of 4 time groups.

	Time (HALO) of sacrifice (mean±SD)				
Parameters	4 HALO	10 HALO	16 HALO	22 HALO	P value
**PER1 mRNA**	4.09±0.18	1.00±0.00	7.17±0.78	4.98±0.09	0.000
**MMP-2 mRNA**	2.47±0.10	3.89±0.07	1.00±0.00	1.60±0.06	<0.001
**Tumor weight(g)**	0.21±0.02	0.24±0.02	0.17±0.02	0.19±0.02	0.001
**PI(%)**	41.67±4.27	42.72±8.04	36.46±0.39	36.55±0.29	0.27
**MI**	3.91±0.23	4.87±0.16	1.93±0.24	3.01±0.21	0.000

### Circadian variations of tumor weight and MI, PI of tumor cells of tumor bearing mice

There was significant difference in the variation of tumor weight of 4 time groups (F = 40.35, P<0.01, listed in Table 2). The single cosine test confirmed that the variation of tumor weight obeyed a circadian rhythm (F = 28.26, P<0.01). Cosine fitted curve of tumor weight was shown in Figure 7B, the peak value of which appeared at 7.51 HALO (the corresponding weight was 0.24 g), whereas the trough value appeared at 19.18 HALO (the corresponding weight was 0.17 g).

Circadian variations of PI of tumor cells of 4 time groups were listed in Table 2. One-way ANOVA analysis indicated that there was no significant difference (F = 1.59, *P* = 0.27). The single cosine test confirmed that the variation of PI of tumor cells obeyed a circadian rhythm (F = 9.66, P = 0.00). Cosine fitted curve of PI of tumor cells was shown in Figure 7C, the peak value of which occurred at 6.63 HALO (the corresponding PI was 43.39%), while the trough value occurred at 18.61 HALO (the corresponding PI was 35.31%).

There was significant difference in the variation of MI of tumor cells of 4 time groups (F = 536.97, P<0.01, listed in Table 2). The single cosine test confirmed that the variation of MI of tumor cells obeyed a circadian rhythm (F = 86.74, P<0.01). Cosine fitted curve of PI of tumor cells was shown in Figure 7D, the peak value of which appeared at 6.91HALO (the corresponding MI was 4.78), whereas the trough value appeared at 18.85 HALO (the corresponding MI was 2.08).

## Discussion

PER1 is an important core clock gene, which regulates proliferations of various cells in human bodies [1], [2], [5], [11]. Aberrant PER1 expression can easily lead to abnormal cell proliferation and the basic characteristic of malignant tumor is its uncontrolled and disordered cell proliferation [10]. Thus, aberrant PER1 expression can easily lead to carcinogenesis. Studies in recent years have indicated that aberrant PER1 expression was highly linked to the carcinogenesis and development of malignant tumors, such as prostate cancer, colon cancer, leukocythemia and breast cancer et al [12]–[17]. However, the expression and function of PER1 differed greatly in various tumor types [5], [12], [13], [15], [17]–[20]. Gery et al [5] demonstrated that PER1 was down-regulated in prostate and colon cancers and played a pro-apoptotic and cancer-suppressive role, while Sato et al [20] showed that PER1 was up-regulated in pancreatic and liver cancers and exerted an anti-apoptotic and cancer-promotive effect. These studies implied that PER1 may play important but different roles in the carcinogenesis and development of different cancers. Our study, for the first time, has demonstrated that PER1 expression was significantly decreased in cancerous tissues compared to that in adjacent noncancerous tissues of patients with BSCC. Advanced analysis showed that decreased PER1 expression was highly correlated with advanced clinical stage and increased risk for regional lymph node metastasis. And there results were opposite to that of MMP-2, which plays an important role in tumor cell invasion and metastasis. That is, MMP-2 expression was increased as PER1 expression was decreased. We suggest that down-regulated PER1 expression was correlated with more advanced cancer stages in patients with BSCC, implying that PER1 is an anti-oncogene and its expression may mainly correlate with the invasion and metastasis of BSCC cells. The results of PER1 expression in BSCC and its correlations with patients' clinical pathologic parameters indicated that PER1 expression might be used to evaluate the stage and metastatic risk of patients with oral squamous cell carcinoma. It might also provide novel ideas for PER1 gene-targeted treatment of oral squamous cell carcinoma.

In recent years, studies [12]–[17] have indicated that aberrant PER1 expression was highly linked to the carcinogenesis and development of malignant tumors, such as breast cancer, colon cancer and leukocythemia. Moreover, the circadian rhythm of PER1 expression varied greatly in different tumor types. Bjarnason et al [23] reported that PER1 mRNA showed a circadian rhythm in healthy human buccal mucosa. The dominant peak value of PER1 mRNA expression was in early activity phase (07:58 h), and the trough value in the transition of activity and rest phase (00:00 h), displaying a approximate 3.33-fold peak-to-trough variation. We, for the first time, studied circadian expression of PER1 in buccal squamous cell carcinoma cells, which demonstrated that PER1 mRNA expression had circadian rhythm, with peak value occurred in middle to late activity phase (19.48 HALO), trough value in middle rest phase (7.64 HALO), showing a approximate 3.81-fold peak-to-trough variation. Though rodents are nocturnal animals and show opposite rest-activity phase compared to human beings, it has been confirmed that results obtained from the rest-activity phase of rodents could extrapolate to the corresponding rest-activity phase of human beings [24]. Therefore, our results demonstrated that while compared to that in healthy human buccal mucosa, the circadian rhythm of PERl mRNA expression in tumor tissues of BSCC had changed.

Uncontrolled cell proliferation, disordered growth and cell invasion and metastasis are the basic characteristics of malignant tumor. MI, PI of tumor cells and tumor weight are significant parameters to reflect cell division, proliferation and tumor growth. And MMP-2 is a typical factor in cancer cell invasion and metastasis [25], [26]. Our study, for the first time, demonstrated that PER1 and MMP-2 expression, MI of BSCC cells and tumor weight had significant differences among 4 time groups. PER1 expression, MI, PI of BSCC cells and tumor weight confirmed to circadian rhythms. Further analysis revealed that MMP-2 expression, MI, PI of tumor cells and tumor weight had negative correlation with PER1 expression. PER1 expression was the maximum while MMP-2 expression MI, PI of tumor cells and tumor weight were the minimum at 16 HALO; while at 10 HALO PER1 expression was the minimum but MMP-2 expression MI, PI of tumor cells and tumor weight were the maximum. Single cosine analysis, which reflects circadian variation, showed peak value of PER1 mRNA expression and trough value of MI, PI of tumor cells and tumor weight all occurred in middle activity phase; whereas trough value of PER1 mRNA expression and peak value of MI, PI of tumor cells and tumor weight all occurred in middle rest phase. These results indicated up-regulated PER1 expression suppressed cell division, proliferation, tumor growth, invasion and metastasis of BSCC, in which PER1 acted as an anti-oncogene. Therefore, PER1 gene may be used as a novel target for the treatment of BSCC. Meanwhile, the above results also indicated that the time factor should be taken into consideration in efficacy evaluation of oral squamous cell carcinoma therapy. For example, efficacy evaluation of various anticancer agents should be performed at the same time points during a day to avoid the differences of results detected at different time points.

The specific mechanism underlying aberrant expression and altered rhythm of PER1 leading to tumorigenesis and tumor development still needs further investigation. Bjarnason GA and Zieker D et al [23], [27] have demonstrated that in healthy human oral mucosa the expression of clock gene PER1 could significantly influence the expressions of cell-cycle-related proteins and tumor-related genes, such as cyclin E,cyclin B, cyclin A, p53, c-Myc and so on. Gery et al [5] investigated PER1 in colon cancer HCT116 cells in vivo, finding that PER1 regulated cell cycle proteins, such as Wee-1, Cyclin B1, Cdc2 and so on. Down-rugulated PER1 modulated cells to pass G2/M checkpoint more easily, which led to a significant increase in the cell proliferation. Yeh KT et al [14], [28] reported that the mechanism of decreased PER1 expression in endometrial carcinoma and breast cancer tissues was that cytosine-phosphate guanosine (CpG) sites promoter methylation of the PER1 gene and this lead to downregulation and inhibition of PER1 expression. Therefore, in this study, we presumed that methylation occurred in PER1 gene promoter in BSCC cells and this resulted in the variation of PER1 expression. As PER1 gene is not only important in regulating cell proliferation, but also in regulating circadian rhythms of living subjects [1], [2], [5], [11], the variation of PER1 expression could not only lead to the circadian changes of oral squamous cell carcinoma cells, but also lead to the variations of MMP-2 expression, PI and MI, by regulating down-streamed cell-cycle-related proteins and tumor-related genes. Therefore, PER1 played a significant part in the occurrence and development of oral squamous cell carcinoma. However, advanced and in-depth studies are still needed to explore the concrete mechanism.

In conclusion, our study demonstrated that the expression of clock gene PER1 was significantly down-regulated in cancerous tissues compared to adjacent noncancerous tissues of patients with BSCC and decreased PER1 expression was correlated with more advanced cancer stages. We also found that PER1 expression, MI, PI of BSCC cells and tumor weight all confirmed to circadian rhythms. PER1 expression had negative correlation with MMP-2 expression, MI, PI of tumor cells and tumor weight. Therefore, studies on expressions and circadian variations of clock genes in different tissues and tumors will help to reveal the mechanism of tumorigenesis and tumor development, which also have great significance in tumor diagnosis, targeted therapy and efficacy evaluation. Meanwhile, it may become a novel method for treating tumors by regaining normal circadian expressions of clock genes in tumor tissues.

## Materials and Methods

### Ethics Statement

This study was approved by the Biomedicine Ethics Committee of the First Affiliated Hospital of Chongqing Medical University and a written informed consent was obtained from each patient prior to tissue acquisition. All animal experimental procedures were performed with approval from the Ethics Committee of Chongqing Medical University.

### Clinical data

Cancerous and adjacent noncancerous tissues were obtained from 38 patients with BSCC, undergoing primary tumor resection in conjunction with neck dissection at the Department of Oral and Maxillofacial Surgery, the First Affiliated Hospital of Chongqing Medical University (Chongqing, China) from September, 2010 to January, 2012. All samples were obtained between 11:00 and 14:00 hours. All the patients were pathologically diagnosed of BSCC and had no preoperative radiotherapy, chemotherapy or other treatments. They were aged 32–79 years (mean age 52.3 years). Clinical pathologic characteristics, including patients' sex, age, smoking status, alcohol use status, areca nut chewing and TNM staging, tumor differentiation, and lymph node metastasis are listed in Table 1. Immediately after resection, each specimen was divided into 2 parts, with one part fixed in 4% paraformaldehyde and embedded in paraffin blocks and the other part snap-frozen in liquid nitrogen and stored until use.

### Cell culture and experimental animals

The human BSCC cell line BcaCD885 (West China School of Stomatology, Sichuan University) cells were cultured in RPMI 1640 with 10% fetal bovine serum (FBS) at 37°C in a humidified atmosphere of 95% air and 5% CO2.

Sixty specific pathogen free (SPF) BALB/c nude mice (male, 5∼6 w, 19∼20 g) were purchased from the Experimental Animal Center of the Chongqing Medical University.

### Establishment of a mouse model of BSCC

Sixty nude mice were randomly housed in separate cages (5 mice per cage). Before being used in studies, mice were housed for 3 weeks under 12 h light-12 h dark cycles (at a room temperature of 24±1°C and humidity of 60±10%). The bedding, food and water of mice were all sterilized. In the 12 h light-12 h dark cycles, time was expressed as hours after light onset (HALO). “0 HALO” was set as the time to turn on the light and “12 HALO” as the time to turn off the light. After 3 weeks, BcaCD885 cells growing at exponential phase were trypsinized with 0.25% of trypsin, centrifuged by frozen low-speed centrifuge (Z233MK-2, HERMLE, Germany) for 5 min (1,000 r/min, 4°C) and resuspended in sterile phosphate buffered saline (PBS). 0.2 mL of PBS containing 2×10^6^ BcaCD885 cells were injected under the oral mucosa of nude mice to establish a nude mice model of BSCC. Afterwards, mice continued to be housed under 12 h light-12 h dark cycles. After 3 weeks noticeable tumors were present. During a period of 24 h, fifteen mice were sacrificed by cervical dislocation at 4 time points including 4 HALO, 10 HALO, 16 HALO and 22 HALO, respectively, and tumors were excised immediately after sacrifice. After being washed with PBS and dried on filter paper, tumor tissues were weighed with a precise balance. Then each tumor was divided into 4 parts, with one part fixed in 4% paraformaldehyde and embedded in paraffin blocks, tumor cells of one part suspended and the other two parts snap-frozen in liquid nitrogen.

### Immunohistochemistry (IHC)

4% paraformaldehyde fixed and paraffin embedded specimen sections (4 µm) were prepared. After dewaxed in xylene and rehydration in graded ethanols, endogenous peroxidase activity was blocked by incubation with 3% H_2_O_2_ at 37°C for 10 minutes. The slides were then heated in a microwave oven twice for 5 minutes in citrate buffer (pH 6.0) at high power to retrieve antigens. After blocking with goat serum at 37°C for 30 minutes, the sections were incubated with the rabbit polyclonal anti-PER1 antibody (primary antibody) (1∶500, Abcam, UK) and the rabbit polyclonal anti-MMP-2 antibody (primary antibody) (1∶1000, Abcam, UK) overnight at 4°C, respectively. After a brief wash with PBS, biotinylated secondary antibody was added at 37°C for 30 minutes according to the manufacturer's recommended protocol (SP kit; ZYMED, USA). Finally, slides were stained using 3, 3′-diaminobenzidine H_2_O_2_, counterstained with hematine, and examined under a microscope. Five high-power fields were examined randomly in each section. Appropriate positive and negative controls were also included. For positive controls, sections provided by the company were stained for PER1 and MMP-2, respectively. Negative controls used all reagents except primary antibody. Staining in nucleus and/or cytoplasm of cells was regarded as positive. The results were determined by dual score semi-quantitative method. The percentage of positive cells in a slice was less than 5% scoring 0, 6%–25% scoring 1, 26%–50% scoring 2, 51%–75% scoring 3 and more than 75% scoring 4. The staining intensity of positive cells (0 point no staining, 1 point yellow, 2 points dilute brown, 3 points brown). Both of the above scores were calculated by multiplication to obtain 0 (negative), 1–4 (positive), and >4 (strongly positive). Both positive and strongly positive were grouped together as positive in statistical analysis.

### Western blot

The cells were lysed using lysis buffer (50 mmol/L Tris-HCl, Ph = 7.4, 150 mmol/L NaCl,0.5%NP40) and centrifuged for 2 min 1,000 r/min, 4°C. The protein concentration was determined using the bicinchoninic acid (BCA) assay. The lysates (50 µg protein) were subjected to SDS–PAGE and the proteins were transferred to PVDF membranes. The membranes were incubated with rabbit polyclonal anti-PER1 antibody (1∶200) and anti-Tubulin antibody (1∶1,000, Beyotime, China) overnight at 4°C, followed by peroxidase-conjugated AffiniPure goat anti-rabbit IgG (1∶5,000) and peroxidase-conjugated AffiniPure goat anti-mouse IgG (H+L) (1∶5,000, both from Zhongshan goldenbridge biotechnology, China) at 37°C for 60 min. The ECL-advance Western Blot Detection System was used for detection (ChemiDocXRS+, Bio-Rad, USA).

### Real-time RT-PCR

The experimental procedure was described as follows. Briefly, (1)RNA extraction. Total RNA from tissues was extracted using RNAiso Plus (Takara, Japan) and RNA concentration and quality was assessed by UV/Visible Spectrophotomete (GeneQuant, Amersham Biosciences, Sweden). (2) cDNA preparation. cDNA was synthesized by reverse transcription from 20 µl of total RNA using PrimeScript RT regent Kit (Takara, Japan) under the conditions of 15 min at 37°C, followed by 5 s at 85°C on a reverse transcription machine (1000TM Thermal Cycler, Bio-Rad, USA). (3)Real-time RT-PCR. Specific primers of β-actin, a housekeeping gene acted as internal control, and PER1 and MMP-2 gene were designed using Oligo17.0 software. Their sequences and product sizes were listed in Table 3. PCR reactions containing 12.5 µl of 2×SYBR Premix Ex TaqTM II (Takara, Japan), 8.5 µl of RNAse free water, 1 µl of 0.4 µM forward primer and reverse primer, respectively, and 2 µl of cDNA in a total volume of 25 µl, were amplified on a real time PCR machine (C-1000TM Thermal Cycler, Bio-Rad, USA) using SYBR Premix Ex Taq II, Perfect Real Time. Cycling conditions were as follows: 1.5 min at 95°C followed by 40 rounds of 10 s at 95°C, 30 s at 60°C. Data were acquired as threshold cycle (Ct) value. The relative PER1 and MMP-2 mRNA expression in tumor cells was calculated using the 2^−ΔΔCt^ method. Each sample was performed in triplicate to ensure the accuracy of the data.

**Table 3:** The sequences and product sizes of PER1, MMP-2 and β-actin used in real-time RT-PCR.

Gene	Primer	Primer sequences	Product size(bp)
**PER1**	forward primer	5′-ACCCTGATGACCCACTCTTCTC-3′	170
	reverse primer	5′-CTCCTCCATAGCCAAGTCCTGA-3′	
**MMP-2**	forward primer	5′-ATTCCGCTTCCAGGGCACAT-3	159
	reverse primer	5′-GCACCTTCTGAGTTCCCACCAA-3′	
**β-actin**	forward primer	5′-CTTCTACAATGAGCTGCGTGTG-3′	169
	reverse primer	5′-AGAGGCGTACAGGGATAGCACAG-3′	

### Proliferation index assay

The experimental procedure was briefly described as follows: After washed 3 times with D-Hanks solution, tumors were treated with penicillin (5×10^5^ U/L) and streptomycin (100 mg/L) for 20 min. Then tumors were cut into small pieces (about 1 mm×1 mm×1 mm) and immersed in digestive solution (2 g/L of collagenase, 1 g/L of DNase, and 0.0002 g/L of Hyaluronidase) for 40 min and filtered with 200-mesh tantalum net. The filtered solutions were centrifuged for 5 min (1,000 r/min, 4°C) and the cell pellets were washed three times with PBS and then resuspended in RPMI 1640 medium. All cell concentrations were adjusted to 1×10^6^/mL and 1 mL of cell suspension solution was centrifuged for 5 min (1,000 r/min, 4°C). The cell pellets were fixed in 70% ethanol at −20°C and then kept at 4°C overnight. The cells were spun down by centrifuging for 5 min (1,000 r/min, 4°C), followed by 2 washes with PBS and 30 min staining with Propidium iodide solution at 4°C in the dark. The following formula was applied to calculate the proliferation index (PI) of tumor cells using flow cytometer (FACSVantage, BD, USA): PI = (S+G2/M)/(G0/G1+S+G2/M)×100%. (G0, G1, G2, S and M correspond to the different phases of the cell cycle). The experiment was conducted in triplicate to ensure the accuracy.

### Mitotic index assay

One hematoxylin and eosin stained (HE) section was prepared from each tumor and evaluated under microscope (BX51, Olympus, Japan). Areas containing the most viable tumor cells were chose for imaging. Five consecutive images (approximately 300 to 400 tumor cells per image) were randomly taken from each section using ×400 magnification. The images were viewed on computer screen using Adobe Photoshop software and the numbers of mitotic figure in each image were counted. The average mitotic figures from five images (mitotic index) from each tumor were calculated.

### Statistical analysis

Calculations were performed with the SPSS 13.0 statistical software package. For numerical data, mean and SD were calculated and graphed. The association between PER1 expression and patients' clinical pathologic characteristics was analyzed using Fisher probabilities test. One-way ANOVA was used to analyze the differences of the 4 time groups. Circadian rhythm was assessed by single cosine test using CircWave 1.4 software (Holland). A P value<0.05 was required for statistical significance.
